# Theoretical Mechanisms of GLP-1 Receptor Agonist-Induced Depression in Obesity Treatment

**DOI:** 10.1192/j.eurpsy.2025.411

**Published:** 2025-08-26

**Authors:** A. Stewart, B. Carr

**Affiliations:** 1 College of Allopathic Medicine, Nova Southeastern Univeristy, Ft. Lauderdale; 2 Psychiatry, University of Florida, Gainesville, United States

## Abstract

**Introduction:**

Recent post-marketing data reports an increased incidence of depression and suicidal ideation in patients using glucagon-like peptide-1 receptor agonists (GLP-1 RAs) for diabetes and obesity management compared to metformin. Despite ongoing FDA evaluations finding no causal link, various studies present mixed findings and propose several biologically plausible mechanisms for GLP-1 RA-induced depression. Increased depression rates observed post-bariatric surgery suggest similar outcomes following significant weight loss with GLP-1 RAs. This review explores these mechanisms to provide a comprehensive understanding and guide clinical practice.

**Objectives:**

To evaluate theoretical mechanisms underlying GLP-1 RA-induced depression in patients undergoing obesity treatment and to offer insight for later clinical decision making.

**Methods:**

A comprehensive literature review was conducted using PubMed and MEDLINE, alongside current FDA updates. The review focused on GLP-1 RA side effects related to depression and included case reports of depression in patients on GLP-1 RA therapy. Data on incidence and clinical management were synthesized to develop a cohesive understanding of associated risks and recommended practices.

**Results:**

The review identified several theoretical mechanisms through which GLP-1 RAs may induce depression. Firstly, GLP-1 RAs may reduce food cravings by altering hedonic tone, potentially diminishing pleasure from various activities. Secondly, rapid changes in gut microbiota may lead to gut-brain axis dysregulation, contributing to depressive symptoms. Thirdly, GLP-1 RAs have been linked to reduced absorption of vitamin B-12 and other essential nutrients, impacting brain function and mood. Social media data and individual case reports reveal mixed mental health outcomes, with some users reporting improved mood and others experiencing mood deterioration.

**Conclusions:**

The evidence on GLP-1 RAs and depression is mixed. While some studies suggest negative mood effects, others, including the FDA’s large-scale evaluations, have not established a causal link. Notably, semaglutide may be associated with a lower risk of suicidal thoughts. These findings emphasize the complexity of this issue and the necessity for ongoing monitoring and personalized patient care. Regular mental health screenings for patients on GLP-1 RAs, especially those with a history of depression, are recommended. Addressing potential nutrient deficiencies through dietary adjustments or supplements is crucial. Patients should be informed about potential mood changes and encouraged to report any adverse effects. Further research, particularly more longitudinal and large-scale studies, is needed to clarify the relationship between GLP-1 RAs and mental health outcomes.

**Disclosure of Interest:**

None Declared

